# Supplementary material for: The role of health literacy in the relationship between mothers’ knowledge and practices of iron supplementation in children (aged 12 to 24 months): A structural equation model
Source: PLoS One. 2025 Apr 3;20(4):e0319845. doi: 10.1371/journal.pone.0319845 (PMC11967935; doi:10.1371/journal.pone.0319845)
Supplement: Attachment — Submitted filename: point by point.docx [file pone.0319845.s001.docx]

**Dear Editor and reviewers**

**Thank you for the time and attention and the opportunity you have given us to resubmit a revised copy of this manuscript. All the comments and responses are in the below table. Corrections are highlighted in the revised version of the manuscript.**

| Comments | Response |
| --- | --- |
| Reviewer 1 | |
| The manuscript is well-written and presents important insights. To further enhance its quality and impact, the following suggestions are offered:  1. Consider incorporating information on health literacy, such as mental health literacy (e.g., DOI: 10.56101/rimj.v3i1.91) and literacy related to specific diseases (e.g., DOI: 10.56101/rimj.v3i1.75), from other countries. This addition would make the literature review more comprehensive | Thank you, respected reviewer, for encouraging feedback.  I added one of these researches in my literature review. The new reference has been highlighted. |
| 2. Clearly outline how this study builds on existing research and addresses knowledge gaps, particularly regarding the mediating role of health literacy in mothers' knowledge and practices of iron supplementation. | Thank you for the precious comment.  Done. |
| 3. Broaden the discussion of findings in comparison to previous studies. Highlight any unique insights or significant discrepancies to provide a richer context. | Thank you for the precious comment. |
| 4. Expand on the implications of health literacy for broader public health strategies, especially in the context of other nutritional interventions. | Thank you for the precious comment. I understand your concern. I expanded as you mentioned. |
| 5. Include a more detailed explanation of the study’s limitations, specifically addressing how recall bias and selection bias (resulting from the convenience sampling method) may have influenced the findings. | Thank you for the comment.  I added more explanation according to the comment. |
| 6. Offer specific suggestions for future research that could address these limitations and advance the field | Done. |
| 7. Provide more actionable recommendations based on the findings, such as developing tailored health literacy interventions for underperforming groups. | Thank you for pointing out. I have added the same. |
| 8. Enhance the clarity of tables and figures by including more descriptive captions and offering detailed interpretations in the text. | Done. |
| 9. Elaborate on the sample size calculation and explain how it ensures sufficient statistical power for all analyses performed. | Thank you for the comment. I added explanations. |
| 10. Expand the discussion of questionnaire validation processes beyond Cronbach’s alpha, describing any pilot testing or iterative refinements. | Regarding the knowledge and practice questionnaire, we conducted a thorough literature review to identify existing validated tools and theories relevant to our constructs. We also consulted with domain experts to define content areas and ensure the questionnaire items accurately captured the intended constructs.  This explanation was included in the tools and techniques section:  “**This questionnaire was developed by the researcher for confirmation of its validity and reliability. For face and content validity, the comments and suggestions of 10 experts in medical and health area were taken and the questionnaires were corrected. Then for reliability a pilot study was conducted on 30 people with an interval of one week, and Cronbach’s alpha was determined.** “ |
| 11. Offer a more detailed explanation of the statistical methods used for mediation analysis, emphasizing their rigor and the robustness of the findings. | Thank you for the suggestion. Detailed explanations of the statistical methods were added. |
| 12. Discuss in greater depth how the sample reflects the broader population and what this means for the generalizability of the study’s conclusions. | Thank you for your attention. I tried to expand the discussion with greater depth. However, similar studies on iron feeding in children were limited. |
| 13. Ensure consistent use of technical terms and minimize redundancy, particularly in the Introduction and Results sections, to improve readability and focus. | Thank you for pointing out.  I checked the manuscript to reduce redundancy and corrected it accordingly. |
| Reviewer 2 | |
| This manuscript is well-written and demonstrates a high standard of scholarly work. It meets the criteria for publication and is suitable for acceptance in its current form without the need for further revisions | Thank you so much, respected reviewer, for encouraging feedback. |

Thank you once again.

Regards

Dr.Omidvar
